# Characterization and genomic analysis of JC01, a novel bacteriophage infecting *Cronobacter sakazakii*

**DOI:** 10.1007/s00705-022-05663-9

**Published:** 2022-12-17

**Authors:** Jie Jiang, Guanda Lan, Jinghua Li, Jun Yu, Honglan Huang, Yanbo Sun, Cuiting Xu, Dandan Liu, Yunwei Gong, Chunyan Zhao

**Affiliations:** 1 Department of Pathogenobiology, College of Basic Medical Sciences, Jilin University, 130021 Changchun, Jilin People’s Republic of China; 2 Changchun Center for Diseases Control and Prevention, Changchun, People’s Republic of China

## Abstract

The lytic bacteriophage JC01 was isolated using a strain of *Cronobacter sakazakii* previously isolated from powdered infant formula (PIF). The complete genome sequence of phage JC01 was determined. The double-stranded DNA genome of phage JC01 is composed of 61,736 bp with a G + C content of 58.9%, and it contains 76 putative open reading frames (ORFs) without any tRNA genes. The predicted ORFs were classified into functional groups, including DNA manipulation, transcription, phage packaging, phage structure, host lysis, and hypothetical proteins. Based on overall nucleotide sequence comparisons, calculation of phage intergenomic similarities, and phylogenetic analysis, JC01 appears to be a novel bacteriophage infecting *C. sakazakii*.

**Supplementary Information:**

The online version contains supplementary material available at 10.1007/s00705-022-05663-9.

*Cronobacter sakazakii*, formerly classified as *Enterobacter sakazakii*, was first identified in 1980 [1] and is a Gram-negative, rod-shaped opportunistic pathogen that is present in the environment and in a variety of foods [2, 3]. *C. sakazakii* is closely associated with sporadic cases and outbreaks of necrotizing enterocolitis and meningitis in neonates and infants due to contaminated milk-based powdered infant formula (PIF) [4–6], with case fatality rates as high as 40% [7]. Due to the severity of infections caused by *C. sakazakii*, it is necessary to develop alternative biocontrol agents to prevent *C. sakazakii* contamination in both food processing and production environments.

Bacteriophages (phages) are viruses that infect bacterial cells and are ubiquitous in nature. Phage-based products and their derivatives have gained attention for their antibacterial properties, and several phage-based products have been approved for eliminating food-borne pathogens in the USA [8, 9].

In this study, a novel phage, JC01, was isolated and purified from sewage samples obtained from the sewage treatment system of the First Hospital of Jilin University, Jilin, China. It was shown to have lytic activity against *C. sakazakii*cz-1801. The purified JC01 was propagated to investigate its morphology, adsorption rate, one-step growth curve, thermal and pH stability, and genome characteristics.

To investigate its morphological features, the purified phage stock was loaded onto a copper grid, stained with 2% uranyl acetate, and viewed using a TECNAI G2 F20 S-Twin transmission electron microscope. JC01 has an isomeric head with a diameter of 56 ± 4 nm and a non-contractile tail with a length of 219 ± 7 nm (Fig. 1).

**Fig. 1:**
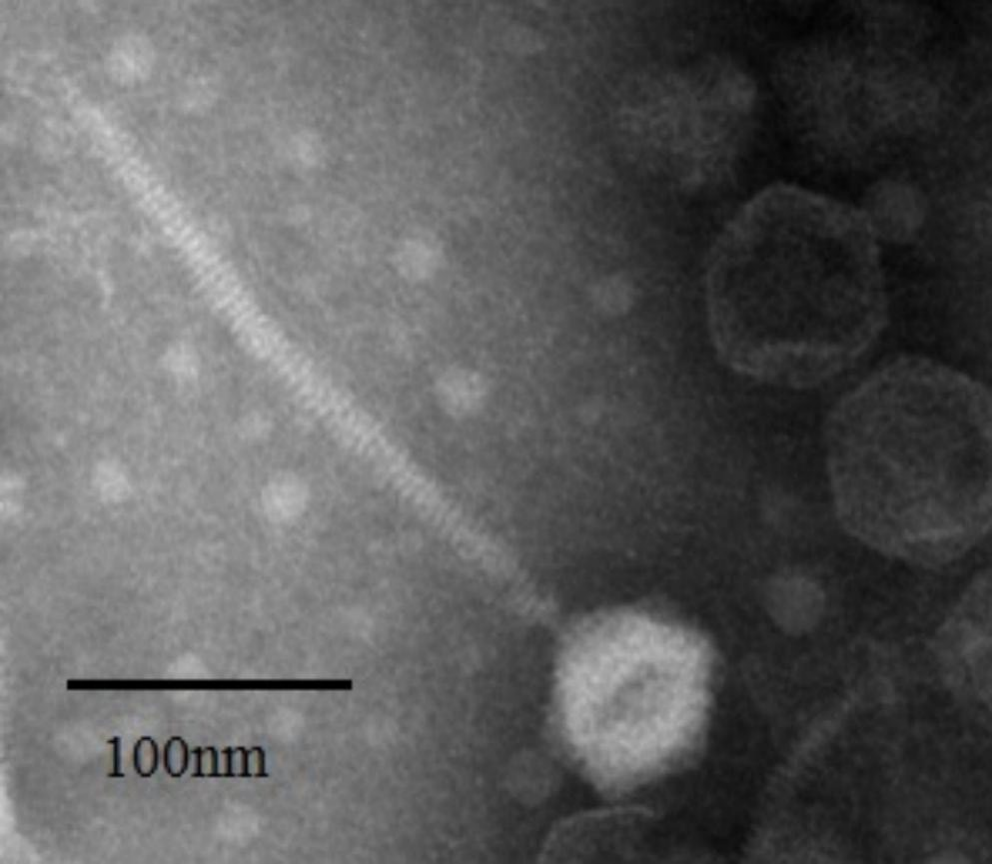
Transmission electron micrograph of *Cronobacter* phage JC01

An adsorption assay was performed as described by Lin and colleagues with some modifications [10]. An aliquot (1 mL) of the phage-host mixture in LB medium at an MOI of 0.01 was collected every 2 min for 20 min. All samples were diluted, and phage titers were determined by the double-layer agar plate method. A one-step growth curve was generated as described by Yang and colleagues [11]. The results demonstrated that approximately 96% of the phage particles adsorbed to the host cells within 14 min (Fig. 2A). The latent and burst periods were approximately 20 and 60 min, respectively. Compared to the previously characterized *C. sakazakii* bacteriophage ФCS01 [12], the latency period of JC01 is 40 min shorter, while the time to reach the burst period is 20 min shorter (Fig. 2B). To investigate the phageʼs ability to survive at different temperatures, the phage suspension was placed at 4°C, 25°C, 37°C, 40°C, 50°C, 60°C, 70°C, and 80°C for 1 h. The phage was also subjected to different pH stresses. The phage suspension was added to LB liquid medium with pH ranging from 3 to 12 at 37°C for 1 h. The results showed that phage JC01 was stable at 4°-40°C and over a wide pH range (5.0–11.0) (Fig. 2C and D). These results suggest that the phage JC01 is active over a wide range of temperatures and would be expected to survive in milk powder manufacturing environments. Therefore, JC01 is a potential biocontrol agent against *C. sakazakii* contamination during food production.

**Fig. 2:**
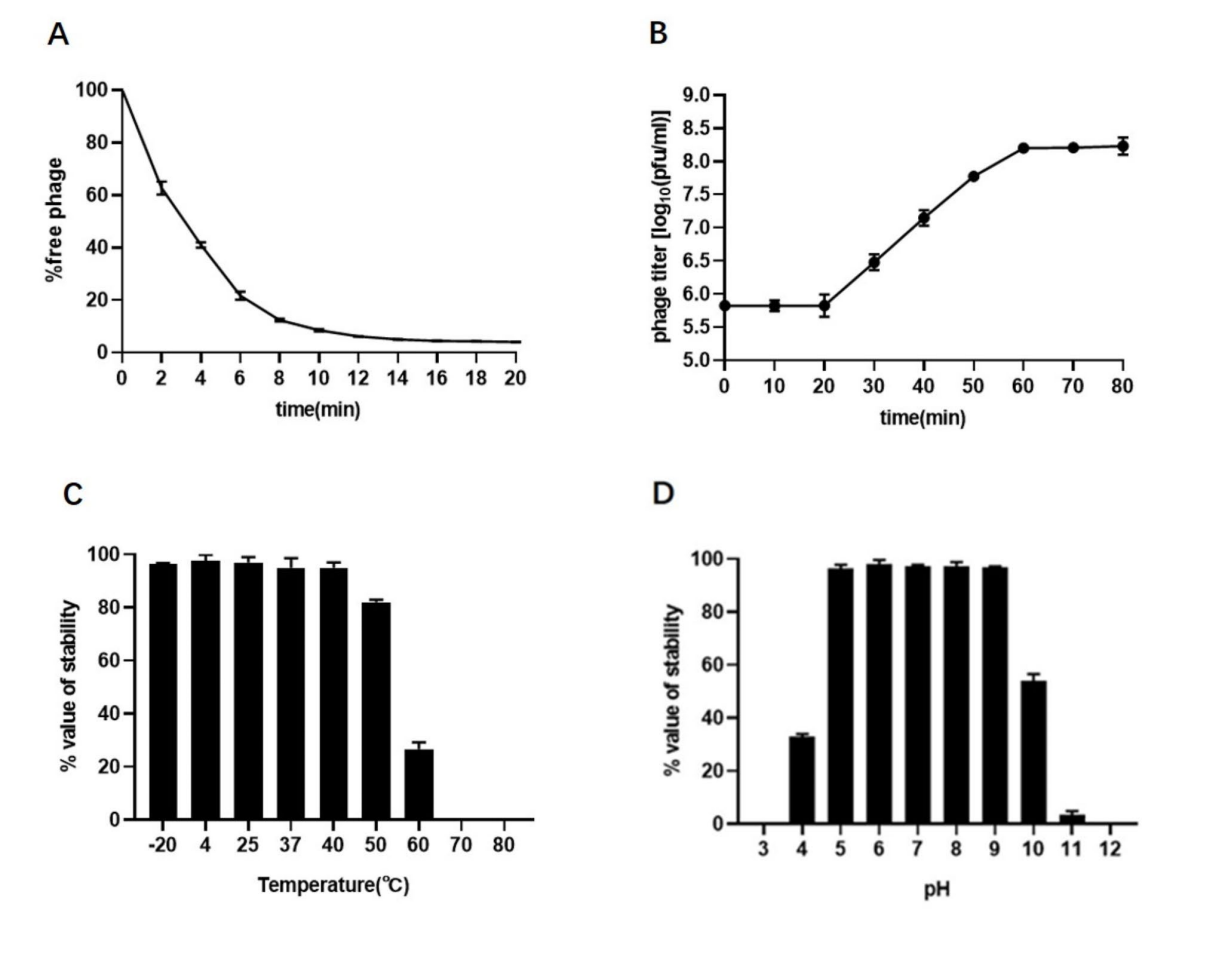
(A) Adsorption assays of phage JC01 with *Cronobacter sakazakii* strain cz-1801. (B) One-step growth curve of phageJC01 in culture broth of *Cronobacter sakazakii* strain cz-1801. (C) Thermal stability of phage JC01. Phages were incubated at different temperatures for 1 h. (D) pH stability of phage JC01. Phages were incubated for 1 h at different pH values. Assays were performed in triplicate, and phage titres are expressed as the mean ± standard deviation.

For genomic DNA analysis, the genomic DNA was extracted from JC01 using the phenol-chloroform method as described previously [13]. Phage DNA was sequenced using an Illumina HiSeq 2500 platform at OE Biotech, China. Raw reads were trimmed to eliminate low-quality reads and adaptor sequences using Trimmomatic [14]. Open reading frames (ORFs) were predicted using GLIMMER [15] and GeneMarks [16]. Possible functions of the encoded proteins were predicted using BLASTp (https://blast.ncbi.nlm.nih.gov/Blast.cgi). Identification of tRNA genes was performed using tRNAscan-SE [17] and RNAmmer (v1.2) [18]. All annotated genes were compared to the comprehensive antibiotic resistance database (CARD) (https://card.mcmaster.ca/) and the virulence factor database (VFDB) (http://www.mgc.ac.cn/VFs/). The complete genome sequence was compared with other nucleotide sequences using BLASTn (https://blast.ncbi.nlm.nih.gov/Blast.cgi). Furthermore, the intergenomic similarities of phages were identified using VIRIDIC (http://rhea.icbm.uni-oldenburg.de/VIRIDIC/) [19]. Phylogenetic trees based on amino acid sequence alignments were constructed in MEGA 7 using the neighbor-joining (NJ) method with 1000 bootstrap replicates [20].

Sequence analysis revealed that phage JC01 contains a double-stranded DNA composed of 61,736 bp with a GC content of 58.9%, with no evident tRNA genes. The genome contains 76 predicted open-reading frames (ORFs), 45 of which are on the plus strand and 31 of which are on the minus strand. The most common start codon in the genome of JC01 is ATG, which is present in 74 ORFs. Only two ORFs have a GTG start codon. The genome has the same terminal 12-base-pair sequence, GGTGCGCAGAGC, that is found at both ends of chi-like phage genomes [21, 22]. Of the 76 ORFs, 26 (34.21%) were predicted to encode functional gene products on the basis of sequence similarity to other phage proteins (e-value < 10^− 3^) in BLASTp searches of the GenBank database, while the other 50 (65.79%) genes were predicted to encode hypothetical proteins. Of the 26 ORFs for which functions were predicted, seven are involved in DNA manipulation, including DNA primase, DNA-binding protein, DNA polymerase I family A, VRR-NUC domain-containing protein, DNA helicase, DNA methyltransferase, and recombination-associated protein. Two ORFs are associated with packaging, encoding the terminase small and large subunits. Thirteen structure- and morphogenesis-related genes of this phage were predicted to encode a head-to-tail joining protein, portal protein, prohead protease, head decorator protein D, major capsid protein E, phage structural protein, pre-tape measure frameshift protein G-T, tape measure protein, four tail assembly proteins, and a tail fiber protein. Three genes were predicted to participate in phage-host interaction and lysis activity: lysis protein B, lysis protein A, and the Rz1 protein [23]. The last of these, a transcriptional regulator, is possibly associated with transcription and translation. The BLAST search results for these proteins are shown in Supplementary Table S1. No antibiotic resistance genes or virulence-factor-related genes were detected in the genome of JC01 using the CARD and VFDB databases.

Bioinformatic analysis using BLASTn showed that the genome sequence of JC01 shares 71.5 to 73.6% identity with six *Salmonella* phages belonging to the genus *Chivirus* of the family *Casjensviridae* (https://talk.ictvonline.org/), suggesting that phage JC01 is distinct from these *Salmonella* phages (Supplementary Table S2). We also calculated the intergenomic similarities between phage JC01 and other known phages by VIRIDIC (Fig. 3). The results showed that JC01 had the highest similarity to *Salmonella* phage FSL SP_124 (KC139515) at 26.8%. According to the guidelines of the International Committee on Taxonomy of Viruses (ICTV), JC01 cannot be classified into any of the currently established genera. To evaluate the evolutionary relationships of the phage, phylogenetic analysis was performed based on the major capsid protein and terminase large subunit sequences, which are highly conserved and used regularly to group phages into families [11, 24]. Both the major capsid protein tree (Supplementary Fig. S1A) and terminase large subunit tree (Supplementary Fig. S1B) revealed that phage JC01 forms a separate branch from the other bacteriophages with sequences in the NCBI database, suggesting that JC01 could have a different ancestor. Thus, it is suggested that JC01 is a novel *Cronobacter* phage.

**Fig. 3:**
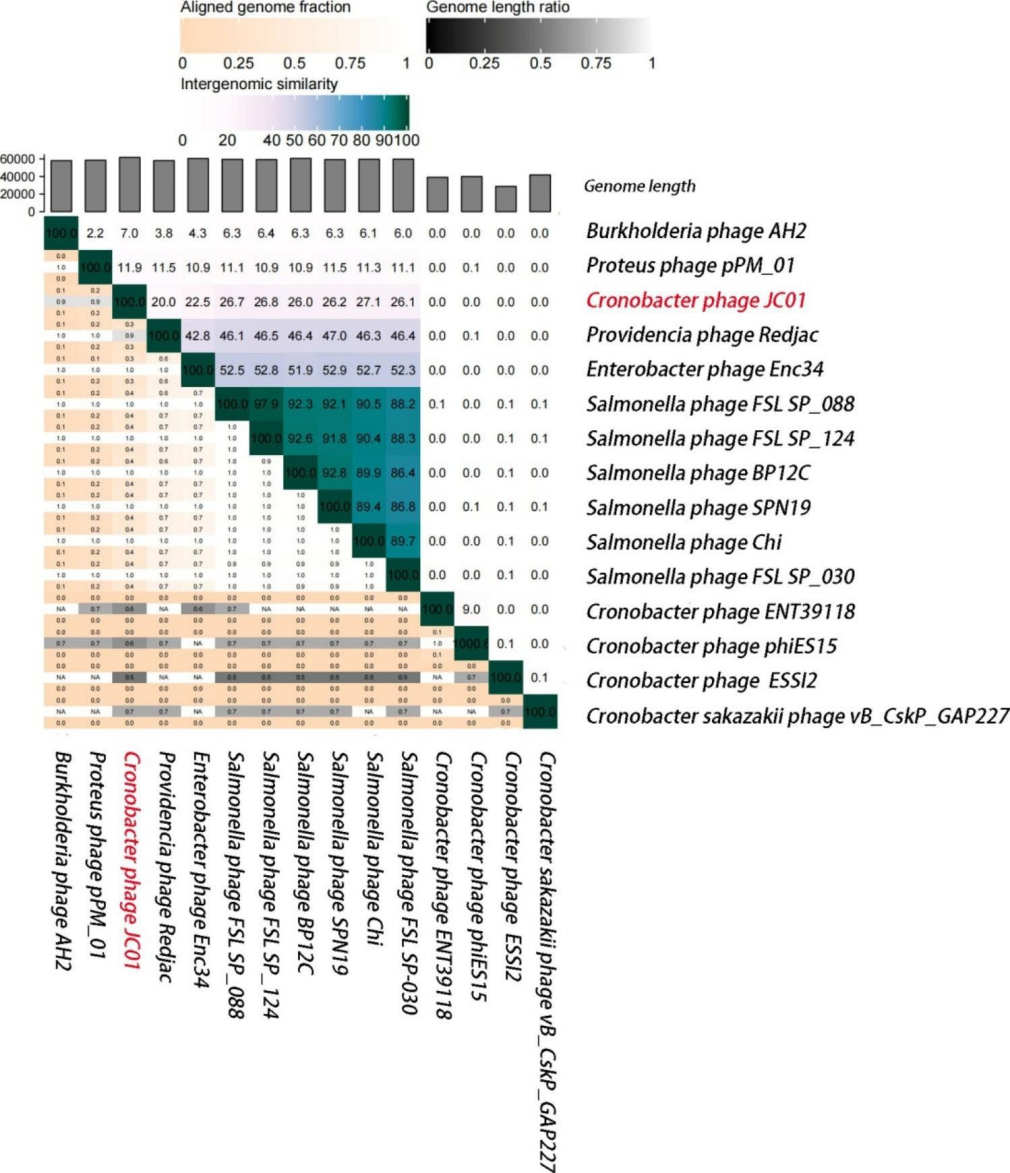
Intergenomic similarities between phages calculated using VIRIDIC. The phage for this study is indicated in red

In order to evaluate the feasibility of phage therapy in food safety, the biological and genetic characteristics of bacteriophages should be comprehensively studied. In this report, JC01 displayed the ability to infect *C. sakazakii* associated with PIF contamination. It remained stable under different environmental conditions and lacked any undesirable genes. Thus, phage JC01 might be a candidate as an alternative biocontrol agent against *C. sakazakii* in food.

**Nucleotide sequence accession number** The complete genome sequence of *Cronobacte*r phage JC01 is available in the GenBank database under accession number MT330372.

## Electronic Supplementary Material

Below is the link to the electronic supplementary material

Supplementary Material 1
