# Supplementary material for: Characterization and genomic analysis of JC01, a novel bacteriophage infecting Cronobacter sakazakii
Source: Arch Virol. 2022 Dec 17;168(1):1. doi: 10.1007/s00705-022-05663-9 (PMC9759504; doi:10.1007/s00705-022-05663-9)
Supplement: Supplementary file 1 — Supplementary Material 1 [file 705_2022_5663_MOESM1_ESM.docx]

**Supplementary materials**

Characterization and Genomic Analysis of JC01, a Novel Bacteriophage Infecting *Cronobacter sakazakii*

Jie Jiang^1^,Guanda Lan^1^,Jinghua Li^1^,Jun Yu^1^, Honglan Huang^1^,Yanbo Sun^1^,Cuiting Xu^1^,Dandan Liu^1^,Yunwei Gong^2^,Chunyan Zhao^1*^

1 Department of Pathogenobiology,College of Basic Medical Sciences,Jilin University,Changchun,Jilin,130021,People’s Republic of China;

2 Changchun Center for Diseases Control and Prevention;

^*^Correspondence auther:Chunyan Zhao

Email:zhaocy@jlu.edu.cn

<Tel:+86> 431 85619574


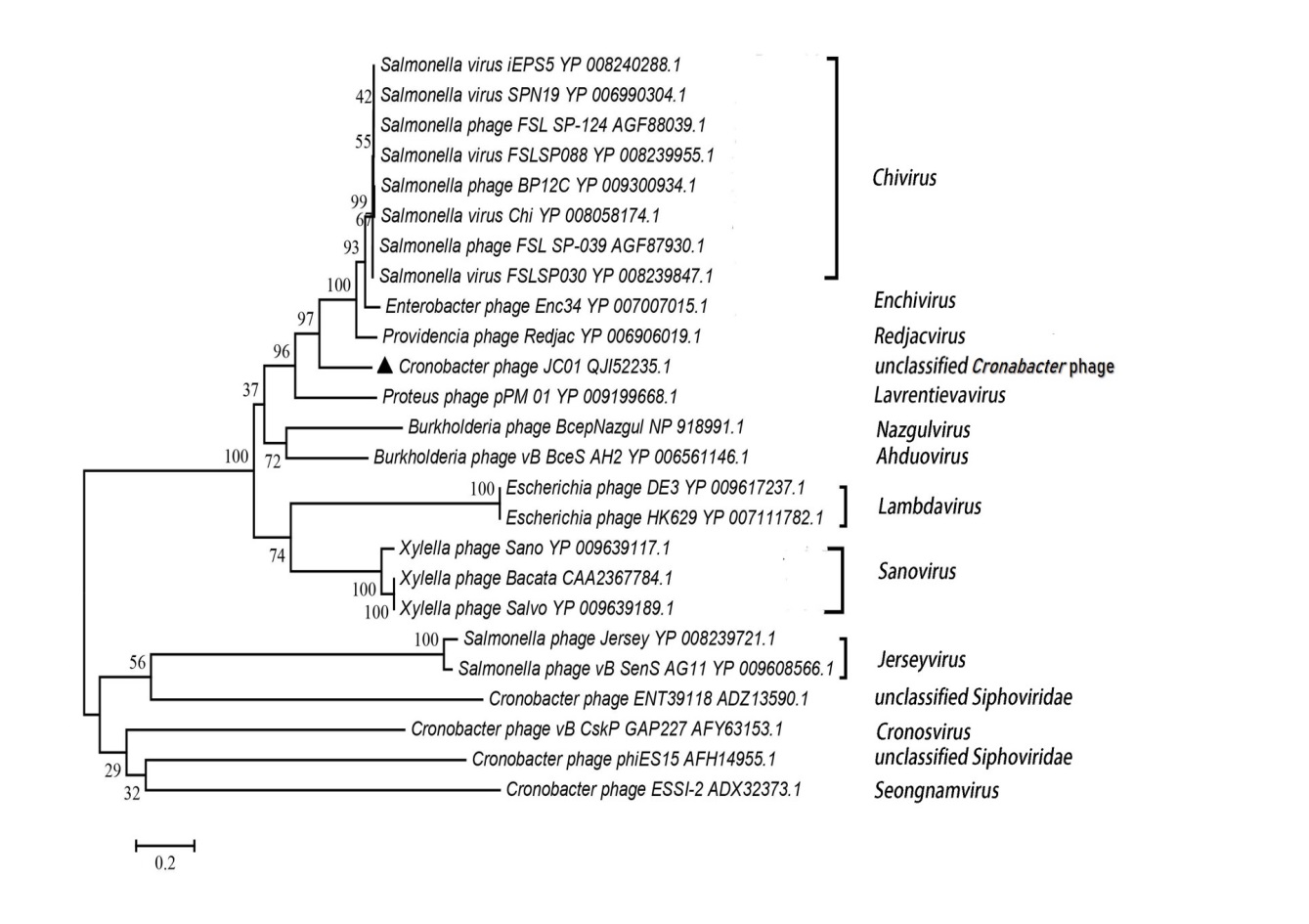


**A**


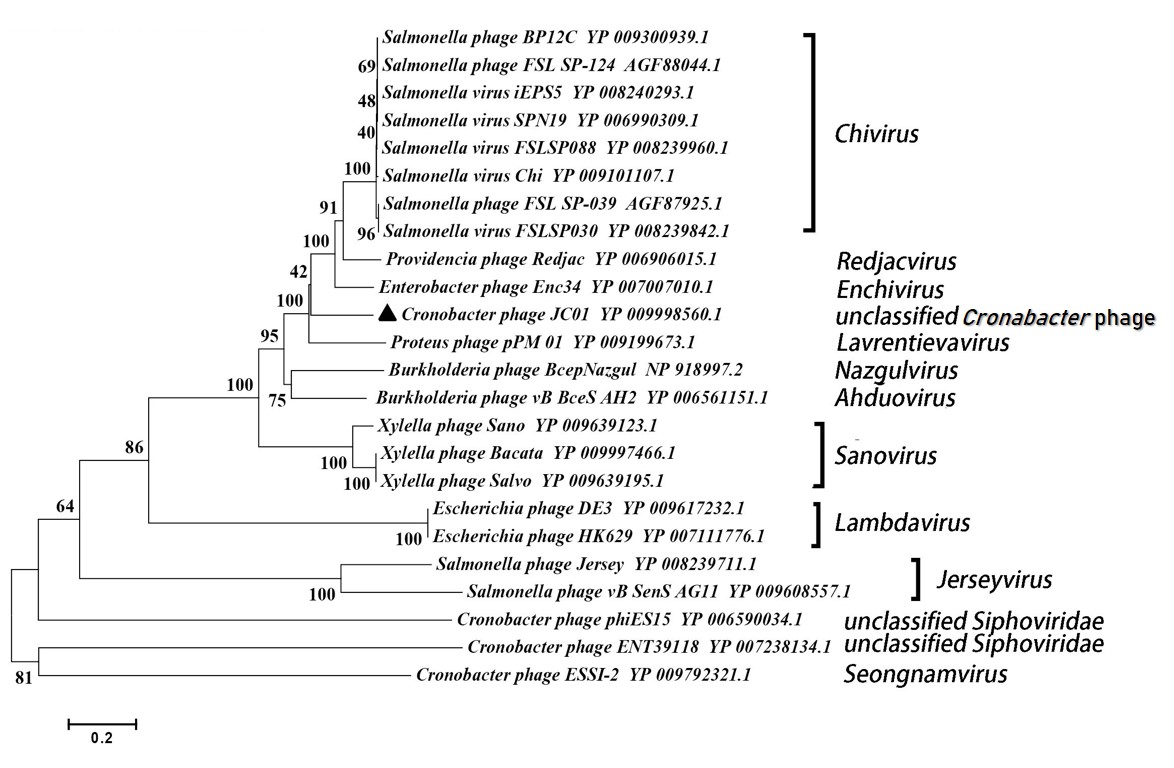


**B**

Supplementary Fig.S1 Phylogenetic analysis of the selected phages based on their major capsid protein (A) and the terminase large subunit (B). The phylogenetic tree was generated in MEGA 7.0 software using the neighbor-joining method.

|  | Supplementary Table 1 ORF analysis of the JC01 genome and conserved domain | | | | | | | | | | | |
| --- | --- | --- | --- | --- | --- | --- | --- | --- | --- | --- | --- | --- |
|  | Label | Strand | Start | Stop | Length  (aa) | Predicted function | Best match | Accession | Query Cover | E-value | Identidy | Conserved domains accession |
|  | gp1 | + | 333 | 437 | 34 | hypothetical protein | - | - | - | - | - | - |
|  | gp2 | - | 549 | 3182 | 877 | DNA primase | DNA primase [Proteus phage Saba] | QEG09449.1 | 98 | 0.00 | 48.19 | cl01287 |
|  | gp3 | - | 3179 | 3460 | 93 | transcriptional regulator | putative transcriptional regulator [Enterobacter phage Enc34] | YP_007007001.1 | 100 | 1.00E-07 | 34.04 | - |
|  | gp4 | + | 3704 | 4081 | 125 | hypothetical protein | hypothetical protein[Pseudomonas phage vB_Pae-SS2019XI] | QIG56952.1 | 64 | 1.00E-09 | 43.75 | - |
|  | gp5 | + | 4121 | 5461 | 446 | hypothetical protein | DNA replication ATP-dependent helicase/nuclease D[Klebsiella phage Soft] | YP_009851425.1 | 98 | 2.00E-141 | 48.75 | - |
|  | gp6 | + | 5520 | 6155 | 221 | DNA binding protein | putative DNA binding protein [Salmonella phage 35] | YP_009830033.1 | 94 | 1.00E-16 | 57.58 | cl29140 |
|  | gp7 | + | 6215 | 8269 | 684 | DNA polymerase I family A | DNA polymerase I family A [Enterobacter phage Enc34] | YP_007007006.1 | 100 | 0.00 | 58.45 | cl02626 |
|  | gp8 | + | 8269 | 8556 | 35 | putativeVRR-NUC domain-containing protein | endonuclease [Proteus phage Saba] | QEG09443.1 | 91 | 0.003 | 59.38 | cl22959 |
|  | gp9 | + | 8597 | 10072 | 491 | DNA helicase | DNA helicase [Enterobacter phage Enc34] | YP_007007008.1 | 95 | 3.00E-176 | 52.1 | cl28899 |
|  | gp10 | + | 10074 | 10667 | 197 | putative terminase small subunit | terminase small subunit [Salmonella phage SAP012] | BCG45225.1 | 99 | 2.00E-80 | 59.69 | cl06347 |
|  | gp11 | + | 10651 | 12723 | 690 | terminase large subunit | terminase large subunit [Enterobacter phage Enc34] | YP_007007010.1 | 96 | 0.00 | 66.32 | cl21617 |
|  | gp12 | + | 12731 | 12985 | 84 | putative head-to-tail joining protein | head-to-tail connector complex protein [Proteus phage Saba] | QEG09439.1 | 95 | 1.00E-22 | 54.88 | cl03738 |
|  | gp13 | + | 12985 | 14637 | 550 | portal protein | portal protein [Escherichia phage Utah] | APD19336.1 | 99 | 0.00 | 65.77 | pfam05136 |
|  | gp14 | + | 14630 | 15994 | 454 | prohead protease | prohead protease ClpP [Salmonella phage FSL SP-019] | AGF89266.1 | 100 | 3.00E-127 | 51.74 | cd07022 |
|  | gp15 | + | 16010 | 16405 | 131 | head decorator protein D | head decoration protein [Klebsiella phage Soft] | QEM42189.1 | 99 | 4.00E-50 | 67.94 | pfam02924 |
|  | gp16 | + | 16417 | 17478 | 353 | major capsid protein E | major capsid protein E [Enterobacter phage Enc34] | YP_007007015.1 | 100 | 0.00 | 66.57 | pfam03864 |
|  | gp17 | + | 17538 | 17942 | 134 | hypothetical protein | hypothetical protein [Enterobacter phage Enc34] | YP_007007016.1 | 35 | 6.00E-10 | 53.19 | - |
|  | gp18 | + | 17945 | 18304 | 119 | hypothetical protein | hypothetical protein[Salmonella phage SAP012] | BCG45217.1 | 99 | 5.00E-36 | 49.15 | - |
|  | gp19 | + | 18307 | 18930 | 207 | hypothetical protein | hypothetical protein SP030_00100 [Salmonella virus FSLSP030] | YP_008239850.1 | 100 | 3.00E-98 | 68.27 | - |
|  | gp20 | + | 18930 | 19422 | 170 | hypothetical protein | hypothetical protein [Enterobacter phage Enc34] | YP_007007019.1 | 94 | 2.00E-64 | 61.82 | - |
|  | gp21 | + | 19443 | 20585 | 380 | phage structural protein | phage structural protein [Providencia phage Redjac] | YP_006906023.1 | 98 | 8.00E-146 | 60.05 | pfam02369 |
|  | gp22 | + | 20688 | 21143 | 151 | pre-tape measure frameshift protein G-T | pre-tape measure frameshift protein G-T[Salmonella virus FSLSP030] | YP_008239853.1 | 96 | 4.00E-33 | 43.14 | - |
|  | gp23 | + | 21375 | 25883 | 1502 | putative tape measure protein | putative tape measure protein [Salmonella virus SPN19] | YP_006990296.1 | 99 | 0.00 | 54.11 | cl31236 |
|  | gp24 | + | 25883 | 27583 | 566 | tail assembly protein | tail assembly protein [Enterobacter phage Enc34] | YP_007007025.1 | 99 | 0.00 | 50.97 | - |
|  | gp25 | + | 27591 | 28409 | 272 | tail assembly protein | tail assembly protein [Enterobacter phage Enc34] | YP_007007026.1 | 99 | 2.00E-128 | 62.73 | cl37077 |
|  | gp26 | + | 28420 | 28650 | 76 | tail assembly protein | putative tail assembly protein 1 [Proteus phage pPM_01] | YP_009199657.1 | 97 | 4.00E-28 | 63.51 | - |
|  | gp27 | + | 28647 | 28868 | 73 | tail assembly protein | tail assembly protein [Enterobacter phage Enc34] | YP_007007028.1 | 97 | 1.00E-23 | 60.56 | - |
|  | gp28 | + | 28852 | 32670 | 1272 | hypothetical protein | hypothetical protein SP030_00150 [Salmonella virus FSLSP030] | YP_008239860.1 | 99 | 0.00 | 47.94 | - |
|  | gp29 | + | 32673 | 33410 | 245 | tail fiber protein | tail fiber protein [Providencia phage Redjac] | YP_006905987.1 | 98 | 4.00E-102 | 59.26 | pfam10983 |
|  | gp30 | + | 33418 | 34371 | 317 | hypothetical protein | hypothetical protein SP088_00195 [Salmonella virus FSLSP088] | YP_008239940.1 | 99 | 2.00E-62 | 37.20 | - |
|  | gp31 | + | 34376 | 35353 | 325 | hypothetical protein | hypothetical protein Redjac_0050 [Providencia phage Redjac] | YP_006905989.1 | 100 | 6.00E-116 | 53.96 | - |
|  | gp32 | + | 35367 | 36392 | 341 | hypothetical protein | hypothetical protein SPN19_040 [Salmonella virus SPN19] | YP_006990287.1 | 99 | 3.00E-166 | 66.96 | - |
|  | gp33 | + | 36402 | 37559 | 385 | hypothetical protein | hypothetical protein pEpSNUABM08_34 [Erwinia phage pEp_SNUABM_08] | QEQ94781.1 | 99 | 2.00E-92 | 44.36 | - |
|  | gp34 | + | 37569 | 38645 | 358 | hypothetical protein | hypothetical protein pEpSNUABM08_35 [Erwinia phage pEp_SNUABM_08] | QEQ94782.1 | 100 | 3.00E-61 | 38.79 | - |
|  | gp35 | + | 38655 | 40763 | 702 | hypothetical protein | hypothetical protein SPN19_038 [Salmonella virus SPN19] | YP_006990285.1 | 99 | 1.00E-105 | 35.44 | - |
|  | gp36 | + | 40807 | 41136 | 109 | putative lysis protein B | putative lysis protein B [Proteus phage pPM_01] | YP_009199647.1 | 88 | 6.00E-18 | 42.71 | - |
|  | gp37 | + | 41139 | 41894 | 251 | lysis protein A | lysis protein A [Salmonella virus FSLSP088] | YP_008239935.1 | 94 | 2.00E-108 | 67.36 | cl30808 |
|  | gp38 | + | 42084 | 42341 | 85 | putative Rz1 protein | putative Rz1 protein [Enterobacter phage Enc34] | YP_007007040.1 | 82 | 2.00E-12 | 50 | - |
|  | gp39 | - | 42434 | 42916 | 160 | hypothetical protein | - | - | - | - | - | - |
|  | gp40 | - | 42909 | 43343 | 144 | hypothetical protein | - | - | - | - | - | - |
|  | gp41 | - | 43340 | 43651 | 103 | hypothetical protein | - | - | - | - | - | - |
|  | gp42 | - | 43648 | 44025 | 125 | hypothetical protein | - | - | - | - | - | - |
|  | gp43 | - | 44022 | 44351 | 109 | hypothetical protein | - | - | - | - | - | - |
|  | gp44 | - | 44422 | 44619 | 65 | hypothetical protein | - | - | - | - | - | - |
|  | gp45 | - | 44631 | 44948 | 105 | hypothetical protein | hypothetical protein pPM01_0028 [Proteus phage pPM_01] | YP_009199641.1 | 64.00 | 5.00E-04 | 36.76 | - |
|  | gp46 | - | 45008 | 45217 | 69 | hypothetical protein | - | - | - | - | - | - |
|  | gp47 | - | 45314 | 46015 | 233 | DNA methyltransferase | DNA methyltransferase [Enterobacter phage Enc34] | YP_007007051.1 | 99 | 6.00E-83 | 54.01 | cl05442 |
|  | gp48 | - | 46005 | 46490 | 161 | hypothetical protein | hypothetical protein pEpSNUABM08_63 [Erwinia phage pEp_SNUABM_08] | QEQ94810.1 | 73 | 2.00E-30 | 55.93 | - |
|  | gp49 | - | 46487 | 46864 | 125 | hypothetical protein | hypothetical protein SP049_00125 [Salmonella phage FSL SP-049] | AGF88946.1 | 92 | 5.00E-34 | 47.01 | - |
|  | gp50 | - | 46848 | 47822 | 324 | hypothetical protein | - | - | - | - | - | - |
|  | gp51 | - | 47928 | 48506 | 192 | hypothetical protein | hypothetical protein [Salmonella phage SAP012] | BCG45184.1 | 39 | 1.00E-07 | 42.86 | - |
|  | gp52 | - | 48516 | 48710 | 64 | hypothetical protein | - | - | - | - | - | - |
|  | gp53 | - | 48713 | 49216 | 167 | hypothetical protein | - | - | - | - | - | - |
|  | gp54 | - | 49216 | 49959 | 247 | hypothetical protein | hypothetical protein SPN19_019 [Salmonella virus SPN19] | YP_006990266.1 | 98 | 6.00E-49 | 39.27 | - |
|  | gp55 | - | 50116 | 50382 | 88 | hypothetical protein | hypothetical protein BP12C_20 [Salmonella phage BP12C] | YP_009300895.1 | 100 | 2.00E-25 | 54.95 | - |
|  | gp56 | - | 50382 | 50645 | 87 | hypothetical protein | hypothetical protein [Enterobacter phage Enc34] | YP_007007058.1 | 81 | 9.00E-18 | 47.89 | - |
|  | gp57 | - | 50590 | 51816 | 408 | recombination-associated protein | recombination-associated protein [Enterobacter phage Enc34] | YP_007007059.1 | 83 | 7.00E-30 | 28.09 | cl01122 |
|  | gp58 | - | 51813 | 52157 | 114 | hypothetical protein | hypothetical protein SPN19_015 [Salmonella virus SPN19] | YP_006990262.1 | 78 | 0.64 | 28.89 | - |
|  | gp59 | - | 52147 | 52407 | 86 | hypothetical protein | - | - | - | - | - | - |
|  | gp60 | - | 52397 | 52630 | 77 | hypothetical protein | - | - | - | - | - | - |
|  | gp61 | - | 52653 | 53003 | 116 | hypothetical protein | - | - | - | - | - | - |
|  | gp62 | - | 52990 | 53376 | 128 | hypothetical protein | - | - | - | - | - | - |
|  | gp63 | - | 53369 | 54034 | 221 | hypothetical protein | - | - | - | - | - | - |
|  | gp64 | - | 54045 | 54581 | 178 | hypothetical protein | exonuclease [Salmonella phage vB_SenS_Sasha] | YP_009787841.1 | 94 | 3.00E-68 | 59.76 | - |
|  | gp65 | - | 54578 | 55435 | 285 | hypothetical protein | - | - | - | - | - | - |
|  | gp66 | - | 55432 | 56208 | 258 | hypothetical protein | hypothetical protein pEpSNUABM08_66 [Erwinia phage pEp_SNUABM_08] | QEQ94813.1 | 21 | 0.001 | 50.88 | - |
|  | gp67 | - | 56274 | 56684 | 136 | hypothetical protein | hypothetical protein [Enterobacter phage Enc34] | YP_007007063.1 | 75 | 1.00E-21 | 44.12 | - |
|  | gp68 | + | 57373 | 57633 | 86 | hypothetical protein | hypothetical protein SP088_00050 [Salmonella virus FSLSP088] | YP_008239911.1 | 98 | 2.00E-39 | 72.94 | - |
|  | gp69 | + | 57644 | 58165 | 173 | hypothetical protein | hypothetical protein chi_030 [Salmonella virus Chi] | YP_008058147.1 | 99 | 1.00E-74 | 63.48 | - |
|  | gp70 | + | 58278 | 58901 | 207 | hypothetical protein | - | - | - | - | - | - |
|  | gp71 | + | 58898 | 59104 | 68 | hypothetical protein | hypothetical protein Utah_070 [Escherichia phage Utah] | APD19391.1 | 94 | 6.00E-17 | 57.58 | - |
|  | gp72 | + | 59091 | 59279 | 62 | hypothetical protein | - | - | - | - | - | - |
|  | gp73 | + | 59448 | 59840 | 130 | hypothetical protein | hypothetical protein CR9_166 [Cronobacter phage CR9] | YP_009015128.1 | 90 | 2.00E-15 | 39.34 | - |
|  | gp74 | + | 59905 | 60102 | 65 | hypothetical protein | - | - | - | - | - | - |
|  | gp75 | + | 60357 | 60620 | 87 | hypothetical protein | hypothetical protein pEpSNUABM08_77 [Erwinia phage pEp_SNUABM_08] | QEQ94824.1 | 100 | 2.00E-27 | 56.32 | - |
|  | gp76 | + | 60708 | 61115 | 135 | hypothetical protein | - | - | - | - | - | - |

Supplementary Table 2 Characteristics of the phage JC01 and 6 other *Salmonella* phages belonging to genus *Chivirus,* family *Casjensviridae*

| Bacteriophage | Genome size | GC content  (bp) | ORFs | tRNA | Identity  (%)* | Coverage  (%)* | Accession  number |
| --- | --- | --- | --- | --- | --- | --- | --- |
| *Cronobacter* phage JC01  *Salmonella* phage BP12C  *Salmonella* phageFSL SP-124  *Salmonella* phage SPN19  *Salmonella* phage FSL SP-088  *Salmonella* phage FSL SP-030  *Salmonella* phage iEPS5 | 61,736  60,606  59,245  59,203  59,454  59,746  69,254 | 58.9  56.4  56.5  56.5  56.5  56.6  56.3 | 76  76  71  72  70  71  73 | 0  0  0  0  0  0  0 | 100  73.6  73.6  73.2  73.1  71.5  73.3 | 100  1  1  4  1  2  1 | MT330372  KM366098  KC139515  JN871591  KC139512  KC139519  KC677662 |

* All phage sequences are compared to JC01.
